# Is the TriNetX Database a Good Tool for Investigation of Real-World Management of Von Hippel–Lindau?

**DOI:** 10.15586/jkcvhl.v11i4.324

**Published:** 2024-12-10

**Authors:** Aaron R. Hochberg, Patrick T. Gomella, Brian Im, Anushka Ghosh, Sohan Shah, Rasheed A.M. Thompson, Kevin K. Zarrabi, Mihir S. Shah, J. Ryan Mark, Joseph K. Izes, Costas D. Lallas, Leonard G. Gomella, Adam R. Metwalli

**Affiliations:** 1 Department of Urology, Sidney Kimmel Medical College, Thomas Jefferson University, Philadelphia, PA, USA;; 2 Department of Medical Oncology, Sidney Kimmel Medical College, Thomas Jefferson University, Philadelphia, PA, USA

**Keywords:** hemangioblastoma, pancreatic neuroendocrine tumors, pheochromocytoma, renal cell carcinoma, Von Hippel–Lindau disease

## Abstract

Von Hippel–Lindau (vHL) is a hereditary disease characterized by the development of benign and malignant tumors across multiple organ systems. It is seen in approximately 1 in 36,000 live births. Given that vHL is a rare disease, studies that seek to characterize vHL are often hampered by small sample sizes. The TriNetX database, which contains data from over 100 million patients, may offer the ability to define and describe a large number of vHL patients. The primary objectives of this study were to describe the prevalence of vHL-associated conditions and investigate clinical outcomes using TriNetX. The secondary objective was to compare the results of this analysis to what has been reported in the published vHL literature. TriNetX was queried to establish a cohort of patients with a diagnosis of vHL. This cohort was then used to define the prevalence of the following conditions: reproductive organ (epididymal and broad ligament) cystadenomas, renal cell carcinoma (RCC), pheochromocytomas, endolymphatic sac tumors (ESLTs), central nervous system (CNS) and retinal hemangioblastomas, and pancreatic neuroendocrine tumor (pNETs). A total of 1232 patients in TriNetX had a recorded diagnosis of vHL. Of this, 34 (6.0% of males) patients had epididymal cystadenoma, 21 (3.4% of females) had broad ligament cystadenoma, 352 (28.6%) had RCC, 251 (20.4%) had pheochromocytoma, <10 had ELST, 171 (13.9%) had CNS hemangioblastoma, 34 (2.8%) had pNETs, and 66 (5.4%) had retinal hemangioma. Compared to the existing literature, vHL and associated conditions are underdiagnosed in TriNetX, suggesting its limited use in studying this disease.

## Introduction

Von Hippel–Lindau (vHL) is an autosomal dominant, hereditary disease that leads to the development of various benign and malignant neoplasms across multiple organ systems. It is characterized by a mutation in the VHL gene, which normally encodes the pVHL tumor suppressor protein, located on the short arm of chromosome 3. While the majority of cases are hereditary, a notable portion of cases are sporadic (1). The incidence of vHL is approximately 1 in 36,000 live births, with near complete penetrance (1, 2).

Common manifestations of vHL include reproductive organ (epididymal and broad ligament) cystadenomas, renal cell carcinoma (RCC), pheochromocytomas, endolymphatic sac tumors (ELSTs), central nervous system (CNS) and retinal hemangioblastomas, and pancreatic neuroendocrine tumors (pNETs) (3, 4). Of these, cerebellar hemangioblastomas most commonly induce the presenting symptoms of vHL (5). Further, CNS hemangioblastomas and RCC are the leading causes of mortality related to the disease (1).

VHL is a complex disease with a broad range of clinical manifestations. As a rare disease, most studies seeking to describe vHL can analyze only a small number of patients or pool data from multiple small studies focusing on a single manifestation. The TriNetX network is a research platform with data from over 110 million patients derived from their electronic health records across various healthcare network organizations. TriNetX is a very useful tool in the undertaking of epidemiological research as it can collect data from various institutions and enhance the generalizability of its findings (6, 7). The network can promote and preserve its robustness through rigorous and consistent data quality checks. The network’s robustness in addition to its large and diverse patient population also highlights its utility in the study of rare conditions such as Von Hippel–Lindau (vHL) disease.

In this study, we aim to investigate the prevalence and clinical outcomes of various vHL manifestations within the large patient cohort available through the TriNetX database and to compare these findings with existing literature on vHL. However, it is important to recognize that the data collected from using the TriNetX network also comes with certain limitations. These limitations include inconsistencies in data entry, as well as coding practices that are unique at the individual physician level and across network-participating institutions. Another limitation of using the TriNetX network is the lack of detailed clinical information. This limitation would be particularly impactful to data collected on the manifestations of vHL or diagnoses that may have occurred at institutions outside of the TriNetX network (6, 7). These limitations were carefully considered during the interpretation of the results from the TriNetX dataset.

## Materials and Methods

### Data source

We searched the TriNetX Research Network, a large, global collaborative network containing data from electronic medical records (EMR) of over 110 million patients from 79 healthcare organizations across four countries. TriNetX reports data coded into patients’ EMRs from up to 20 years before the analysis date and excludes patients experiencing the event before this date. As an important note, if the number of patients in a particular search or group is less than 10, TriNetX, to protect patient privacy, will round up and display the number as 10. Cohorts were constructed using Current Procedural Terminology (CPT), International Classification of Diseases-10 (ICD), International Classification of Diseases for Oncology (ICD-O), SNOMED Clinical Terms, Anatomical Therapeutic Chemical, Veterans Affairs National Formulary, and National Library of Medicine RxNorm codes.

### Study population

**Figure 1:**
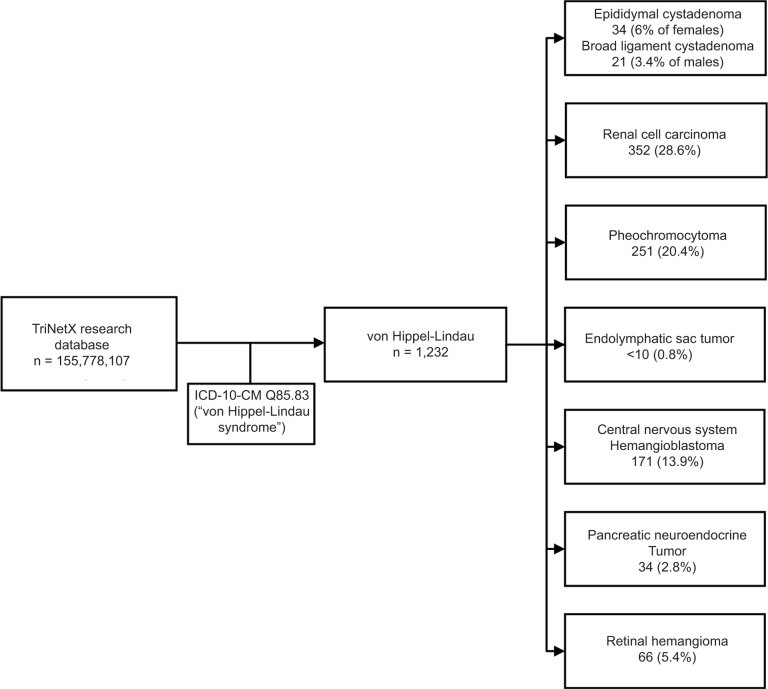
Consort Diagram of vHL diagnoses found within the TriNetX database. Consort diagram showing the prevalence of vHL and vHL-associated conditions in TriNetX. Also shown are criteria for narrowing of TriNetX Research Database to vHL cohort.

Our initial cohort consisted of all patients with vHL (ICD-10 Q85.83). With this cohort, we queried the database for common manifestations of vHL: reproductive organ cystadenomas, RCC, pheochromocytomas, ELSTs, CNS and retinal hemangioblastomas, and pNETs (Figure 1).

### Outcomes and analyses

Selected groups were further analyzed for clinical outcomes and deceased status using descriptive analysis. The RCC cohort was further investigated for the prevalence of radical nephrectomy, partial nephrectomy, renal transplant, and ablation therapy. This cohort was also examined for end-stage renal disease (ESRD) or dialysis status, as well as a diagnosis of chronic kidney disease (CKD) Stages 3–5, and for deceased status. The pheochromocytoma cohort was further investigated for the prevalence of catecholamine storm and deceased status, while the ELST cohort was investigated for the prevalence of surgical treatment and deafness. The CNS hemangioblastoma was further investigated for prevalence of surgical treatment, hemiparesis or hemiplegia, syringomyelia, seizure disorder, intracranial hemorrhage (ICH), and deceased status. The subset of this cohort that underwent surgery was further analyzed for the development of chronic pain after surgery, as well as for death within 6 months of surgery. The pNETs cohort was further investigated for the prevalence of malignant tumors, surgical treatment, pancreatic transplant, insulin dependence, and deceased status. This cohort was also examined for fat-soluble vitamin deficiency, malnutrition, digestive enzyme deficiency requiring exogenous lipase, and osteoporosis/other bone remodeling disorder. The retinal hemangioma cohort was further investigated for the prevalence of blindness, retinal detachment, development of seizures, ICH, and deceased status. A complete list of codes used to query the database is available in Table S1.

We briefly reviewed the available literature to compare the reported prevalence of these conditions with the prevalence observed in TriNetX.

## Results

Our analyses were run on October 17, 2023. Of the 115,778,107 patients in the TriNetX Research Network, a total of 1232 patients had a recorded diagnosis of vHL. Patients were, on average, 36.4 years old at the time of diagnosis, with a standard deviation of 20.2 years and were 41 years old at the time of analysis, with a standard deviation of 20.2 years. The majority of the patients were females (50%) than males (46%), and the remaining (4%) were of unknown sex. The most common race recorded was White (76%), followed by Unknown (12%), Black or African American (5%), and Asian (3%).

Descriptive analysis for the prevalence of vHL-associated conditions was performed on all 1232 patients. It revealed 34 (6.0% of males) patients with epididymal cystadenoma, 21 (3.4% of females) with broad ligament cystadenoma, 352 (28.6%) with RCC, 251 (20.4%) with pheochromocytoma, <10 patients with ELST (0.8%), 171 (13.9%) with CNS hemangioblastoma, 34 (2.8%) with pNETs, and 66 (5.4%) patients with retinal hemangioma.

### Renal cell carcinoma

Further descriptive analyses were performed on selected subgroups. Of the 352 vHL patients with RCC, 44 (12.5%) underwent radical nephrectomy, 95 (27.0%) underwent partial nephrectomy, 23 (6.5%) underwent renal transplant, and 39 (11.1%) patients underwent ablation therapy. Thirty-three patients (9.4%) had ESRD or were on dialysis, and 70 (19.9%) had a diagnosis of CKD Stage 3, 4, or 5. Additionally, 36 (10.2%) patients with vHL and RCC were deceased at the time of analysis.

### CNS hemangioblastoma

Of the 171 vHL patients with CNS hemangioblastoma, 114 (66.7%) patients underwent a neurosurgical intervention, and 12 (7.0%) patients were diagnosed with hemiparesis or hemiplegia. Thirty patients (17.5%) in this cohort had syringomyelia, 21 (12.3%) were diagnosed with some form of seizure disorder, and <10 (5.8%) patients developed ICH. Fifteen patients (8.8%) with vHL and CNS hemangioblastoma were deceased at the time of analysis. Of those who underwent surgery, 43 (37.7%) developed chronic pain after surgery. Less than 10 (8.8%) of the patients died within 3 months of surgery, and there were no deaths between 3 and 6 months after surgery.

### Pancreatic neuroendocrine tumors

Of the 34 vHL with pNETs, 24 (70.6%) patients were diagnosed with malignant pNETs, 11 (32.4%) underwent some form of pancreatic surgery, none underwent pancreatic transplant, and 22 (64.7%) required exogenous insulin. Less than 10 (29.4%) patients were deceased at the time of analysis. Additionally, 14 (41.2%) patients developed a fat-soluble vitamin deficiency and <10 (29.4%) were diagnosed with malnutrition. Fifteen (44.1%) patients were started on an exogenous lipase supplement, suggesting a deficiency of this enzyme. Finally, <10 (29.4%) patients were diagnosed with osteoporosis or other bone remodeling disorder.

### Retinal hemangioma

Of the 66 vHL patients with retinal hemangioma, 19 (28.8%) experienced blindness or low vision and 24 (36.4%) developed retinal detachment. Less than 10 (15.2%) patients were diagnosed with some form of seizure disorder, and there were no patients who developed ICH. Around 15.2% (<10) patients were deceased at the time of analysis.

A comparison of the prevalence of vHL manifestations in this dataset to previously published literature can be found in Table 1. Interestingly, most of these appear to be markedly undercounted in TriNetX, with the notable exception of RCC and pheochromocytoma.

**Table 1:** Review of the literature and TriNetX results on the prevalence of VHL-associated conditions.

Authors	Epididymal Cystadenoma	Broad Ligament Cystadenoma	Renal Cell Carcinoma	Pheochromocytoma	Endolymphatic Sac Tumor	CNS Hemangioblastoma	Retinal Hemangioma	Pancreatic Neuroendocrine Tumor	Reference
Varshney *et al*. (2017)	25-60%	Rare	30%	10–20%	6–15%	60–80%	60%	8% malignant	[1]
Ben-Skowronek & Kozaczuk (2015)						40–80%		2.5% malignant	[3]
Aronow *et al*.(2019)	25-65%	Rare	45%	20%	10–15%	60–80%	45–60%	8–20% overall	[4]
Lonser, Glenn *et al*.(2003)	25-60%	Rare	25–45%	10–20%	11%	60–80%	60%	8–17% overall	[6]
Maher *et al*.(2011)	60%	Rare	70%		11%	60–80%		5–10% overall	[7]
Megerian *et al*.(2002)					11–13%			35–70% overall^a^	[8]
Gioacchini *et al*.(2022)							10%		[9]
Wind & Lonser (2011)			25–45%	10–20%	10–15%	60–80%			[10]
Chew (2005)							50%		[11]
Maher & Kaelin (1997)			24–28%	7–19%	10%	69–72%	57–59%		[12]
Charlesworth *et al*. (2012)								15% overall (12.8% malignant)	[13]
Lonser, Weil *et al*.(2003)						21–72%			[14]
TriNetX	6.0%	3.4%	28.6%	20.4%	0.8%b	13.9%	4.4%	2.8% overall (70.6% malignant)	N/A

a This includes pancreatic cysts and cystadenomas.

b Denotes ≤10 instances in TriNetX.

## Discussion

This study explored the common manifestations and clinical outcomes associated with vHL in a large patient cohort. Given the number of patients in the TriNetX Research Network at the time of analysis (115,778,107) and the commonly accepted incidence of 1 in 36,000 live births, we expected to define a cohort of approximately 3216 patients with vHL. Instead, we observed only 1232 patients with vHL in this large EMR database, less than half the anticipated patient number. Further, the prevalence of vHL-associated conditions aside from RCC and pheochromocytoma was dramatically less than the prevalence reported in the literature (Table 1). We suggest that the reason for this is likely multifaceted but stems from the underdiagnosis of vHL and/or poor EMR coding.

Regarding underdiagnosis, many vHL-associated conditions are not specific to the disease, such as RCC, pheochromocytoma, or certain cystic manifestations. It is plausible that, as a rare disease, vHL is not often considered when these conditions are seen in the absence of other vHL manifestations and further testing is not pursued. Additionally, while vHL has been reported to have up to 90% penetrance, this high degree of penetrance is reportedly not seen until the seventh decade of life. TriNetX may contain a significant portion of younger patients who carry the vHL gene mutation but have not yet been diagnosed with vHL. One study reported that of the known vHL patients who were not undergoing regular, prophylactic surveillance for the development of vHL-associated conditions, as many as 20% of the cohort would have been asymptomatic at age 60 (17). Therefore, there may be a significant number of patients in TriNetX who have as yet undiagnosed vHL.

Underdiagnosis may also be due to problems that exist in how providers establish a diagnosis of vHL. Though vHL is formally diagnosed with genetic testing, guidelines exist for a clinical diagnosis that may suggest the need for further evaluation. One set of criteria, based on Melmon and Rosen’s (18) review, require either a positive family history of the disease plus a single vHL-associated tumor or the presence of two vHL-associated tumors (one of which must be a CNS or retinal hemangioblastoma) in the absence of a confirmed family history (9). Another set of criteria, developed through the use of the Danish vHL database, suggests that a diagnosis may be made if a patient displays any two vHL-associated tumors or if a patient displays one vHL-associated tumor and has at least one first-degree relative with a vHL diagnosis (19, 20). A study conducted using the Danish vHL database offers insight into reasons for failure to successfully record a diagnosis of vHL. These investigators searched national health registries and found that using their clinical criteria, they missed 17% of known vHL patients in this database (17). This suggests that a non-negligible portion of vHL patients do not present in a way that would prompt clinical suspicion, further contributing to underdiagnosis.

Regarding incomplete coding, the aforementioned Danish study also noted that of the patients who did not meet clinical criteria but had known vHL and were in the vHL database, none were assigned the diagnostic code for vHL in the national health registries (15). It is likely that a subset of patients in TriNetX have vHL but do not have their diagnosis coded into their EMR. An additional limitation of EMR coding is that not every vHL-associated condition has a unique ICD-10 code. For example, we could not find a clear and unique ICD-10 code for broad ligament cystadenoma. The lack of specific codes may make it more challenging for a provider to recognize the presence of multiple vHL-associated conditions in a single patient and may fail to pursue additional testing.

Another possible explanation for the paucity of vHL patients in TriNetX is that many vHL patients self-refer to quaternary medical centers that have vHL multidisciplinary care such as the Urologic Oncology Branch of the National Cancer Institute (NCI) at the National Institutes of Health (NIH) or the University of Texas MD Anderson Cancer Center (MDACC) vHL clinic (8, 21, 22). The NIH is certainly not included in the TriNetX dataset since billing and coding are not done for services rendered at that institution. In addition, it is unclear if quaternary centers such as MDACC are included in the TriNetX catchment. Consequently, the sequestration of many vHL patients in these specialized centers may lead to an artificially low representation of vHL patients within this dataset.

The strengths of our study include utilizing a large, multiregional study population to describe a rare disease. This study also has notable limitations, many stemming from the use of the TriNetX database and retrospective reviews, generally. The network is built on diagnostic and procedural codes entered by practitioners into a patient’s EMR. Using TriNetX for any study assumes that the data codes entered are complete and accurate. Several validation studies suggest that EMR coding is accurate (23, 24); however, it is difficult to establish completeness. Additionally, the database does not reveal information on the individual patient, leaving us to infer the relationship between various diagnostic and procedural codes. For example, we can query the database for the number of patients with vHL and RCC who are deceased, but we cannot know that this is the cause of death. Additionally, as previously mentioned, we found that not every vHL-associated condition has a specific ICD-10 code. This forced us to use related codes as a proxy. For the example of broad ligament cystadenoma, we used the broader code ICD-10-CM N83.8 (other noninflammatory disorders of the ovary, fallopian tube, and broad ligament) (see Table S2). With using the TriNetX database, our study is bound by all inherent limitations that come with conducting a retrospective study. These limitations were best mitigated by requiring evidence for vHL diagnosis such as ICD codes and or documented family history. Our study showed the underrepresentation of vHL patients in TriNetX compared to what was expected based on the prevalence seen in other population studies (17). A reason for this observed difference may be attributed to a lack of genetic testing in our study. The Danish database study used a cohort that was genetically confirmed for vHL, whereas our study relied primarily on ICD-10 coding in TriNetX which also did not provide data on genetic testing (17). This could explain the lower number of vHL cases in our study as genetic testing is vital in aiding the diagnosis of cases without classical vHL manifestations. Lastly, while both the Danish study as well as our study identified hallmark manifestations of vHL such as RCC and CNS hemangiomas, in our study we observe fewer patients with multiple vHL associated conditions. This result suggests possible incomplete data capture in the TriNetX network. Overall, our findings in comparison to other large population studies highlight the importance of genetic testing data as well as the need for consistency in diagnostic coding practices. This will serve to promote identification and improved characterization of vHL patients.

Nonetheless, this study was able to complete the objective of characterizing a large cohort of vHL patients, a task that is often impossible for most datasets and institutions. These results could be viewed as a challenge to the existing literature and/or possibly establish a new baseline prevalence of vHL-associated conditions found in regular practice. Furthermore, it is important to have data on clinical outcomes to better educate vHL patients and providers and to better inform treatment options.

## Conclusion

The TriNetX Database does not appear to be well-suited for a detailed study of patients with vHL. The objectives of this study would be better accomplished using a national or international registry of vHL patients. As of the time of submission of this manuscript, no such registry exists in the United States. The vHL Alliance (Boston, MA) is conducting the MyVHL: Patient Natural History Study, which overtime may prove to be a vital asset. While this certainly has some benefits, it may lack in-depth medical information that is desirable for some research. These data highlight the need for a national, clinician-driven vHL database for the further study and characterization of this disease.

## Figures and Tables

**Table S1:** Codes used for VHL and associated conditions.

**VHL**	
ICD-10-CM Q85.83	Von Hippel-Lindau syndrome
**Epididymal Cystadenoma**	
ICD-10-CM D29.3	Benign neoplasm of epididymis
ICD-10-CM N50.3	Cyst of epididymis
**Broad Ligament Cystadenoma**	
ICD-10-CM N83.8	Other noninflammatory disorders of ovary, fallopian tube and broad ligament
**Renal Cell Carcinoma**	
ICD-10-CM C64	Malignant neoplasm of kidney, except renal pelvis
**Pheochromocytoma**	
ICD-10-CM C74	Malignant neoplasm of adrenal gland
ICD-10-CM D35.00	Benign neoplasm of unspecified adrenal gland
ICD-10-CM D35.01	Benign neoplasm of right adrenal gland
ICD-10-CM D35.02	Benign neoplasm of left adrenal gland
ICD-10-CM D49.7	Neoplasm of unspecified behavior of endocrine glands and other parts of the nervous system
**Endolymphatic Sac Tumor**	
ICD-10-CM D14.0	Benign neoplasm of middle ear, nasal cavity and accessory sinuses
**CNS Hemangioblastoma**	
ICD-10-CM D33	Benign neoplasm of brain and other parts of central nervous system
**Pancreatic Neuroendocrine Tumor**	
ICD-10-CM C25.4	Malignant neoplasm of endocrine pancreas
ICD-10-CM D13.7	Benign neoplasm of endocrine pancreas
**Retinal Hemangioma**	
ICD-10-CM D31.2	Benign neoplasm of retina
ICD-10-CM D49.81	Neoplasm of unspecified behavior of retina and choroid
ICD-10-CM D18	Hemangioma and lymphangioma, any site

**Table S2:** Codes used for treatments and outcomes.

**Radical Nephrectomy**	
CPT 50220	Nephrectomy, including partial ureterectomy, any open approach including rib resection
CPT 50234	Nephrectomy with total ureterectomy and bladder cuff; through same incision
CPT 50545	Laparoscopy, surgical; radical nephrectomy
CPT 1008091	Nephrectomy with total ureterectomy and bladder cuff
CPT 1014157	Nephrectomy, including partial ureterectomy, any open approach including rib resection
ICD-10-PCS 0TT00ZZ	Resection of right kidney, open approach
ICD-10-PCS 0TT10ZZ	Resection of left kidney, open approach
**Partial Nephrectomy**	
CPT 50240	Nephrectomy, partial
CPT 50543	Laparoscopy, surgical; partial nephrectomy
SNOMED 81516001	Partial nephrectomy
**Renal Transplant**	
ICD-10-CM	Kidney transplant status
**Ablation Therapy**	
CPT 50592	Ablation, 1 or more renal tumor(s), percutaneous, unilateral, radiofrequency
CPT 50593	Ablation, renal tumor(s), percutaneous, cryotherapy
**Chronic Kidney Disease**	
ICD-10-CM N18.3	Chronic Kidney disease, stage 3 (moderate)
ICD-10-CM N18.4	Chronic Kidney disease, stage 4 (severe)
ICD-10-CM N18.5	Chronic Kidney disease, stage 5
**End Stage Renal Disease/Dialysis**	
ICD-10-CM N18.6	End stage renal disease
CPT 1012740	Dialysis Services and Procedures
**Catecholamine Storm**	
ICD-10-CM E27.5	Adrenomedullary hyperfunction
**Deafness**	
ICD-10-CM H90.5	Unspecified sensorineural hearing loss
ICD-10-CM H91	Other and unspecified hearing loss
**ELST Surgery**	
CPT 69805	Endolymphatic sac operation; without shunt
CPT 69806	Endolymphatic sac operation, with shunt
CPT 1010228	Endolymphatic sac operation
**CNS Hemangioblastoma Surgery**	
CPT 1009068	Surgical procedures on the nervous system
CPT 1009097	Craniectomy or craniotomy procedures
CPT 1009186	Skull base surgical procedures
**Hemiparesis/Hemiplegia**	
ICD-10-CM G81	Hemiplegia and hemiparesis
**Chronic Pain**	
ICD-10-CM R52	Pain, unspecified
ICD-10-CM G89.2	Chronic pain, not elsewhere classified
**Syringomyelia**	
ICD-10-CM G95.0	Syringomyelia and syringobulbia
**Seizures**	
ICD-10-CM G40.89	Other seizures
ICD-10-CM R56	Convulsions, not elsewhere classified
**Intracranial hemorrhage**	
ICD-10-CM I62	Other and unspecified nontraumatic intracranial hemorrhage
**pNET Surgery**	
CPT 48140	Pancreatectomy, distal subtotal, with or without splenectomy; without pancreatojejunostomy
CPT 48150	Pancreatectomy, proximal subtotal with total duodenectomy, partial gastrectomy, choledochoenterostomy and gastrojejunostomy (Whipple-type procedure); with pancreatojejunostomy
CPT 48153	Pancreatectomy, proximal subtotal with near-total duodenectomy, choledochoenterostomy and gastrojejunostomy (pylorus-sparing, Whipple-type procedure); with pancreatojejunostomy
CPT 48155	Pancreatectomy, total
CPT 1007918	Pancreatectomy, distal subtotal, with or without splenectomy
CPT 1007923	Pancreatectomy, proximal subtotal with total duodenectomy, partial gastrectomy, choledochoenterostomy and gastrojejunostomy (Whipple-type procedure)
CPT 1007926	Pancreatectomy, proximal subtotal with near-total duodenectomy, choledochoenterostomy and gastrojejunostomy (pylorus-sparing, Whipple-type procedure)
SNOMED 33149006	Pancreatectomy
**Pancreas Transplant**	
CPT 48160	Pancreatectomy, total or subtotal, with autologous transplantation of pancreas or pancreatic islet cells
CPT 1007944	Pancreas Transplantation Procedures
**Insulin Dependence**	
ATC A10A	Insulins and analogues
VA HS501	Insulin
**Vitamin Deficiency**	
ICD-10-CM E50	Vitamin A deficiency
ICD-10-CM E55	Vitamin D deficiency
ICD-10-CM E56.0	Deficiency of vitamin E
ICD-10-CM E56.1	Deficiency of vitamin K
**Enzyme Deficiency**	
RxNorm 6406	Lipase
**Osteoporosis/Osteopenia**	
ICD-10-CM M80	Osteoporosis with current pathological fracture
ICD-10-CM M81	Osteoporosis without current pathological fracture
ICD-10-CM M85	Other disorders of bone density and structure
ICD-10-CM M89	Other disorders of bone
ICD-10-CM M80.0 (excluded)	Age-related osteoporosis with current pathological fracture
ICD-10-CM M81.0	Age-related osteoporosis without current pathological fracture
**Malnutrition**	
ICD-10-CM E40-E46	Malnutrition
**Blindness**	
ICD-10-CM H54	Blindness and low vision
**Retinal Detachment**	
ICD-10-CM H33	Retinal detachments and breaks
